# *In vitro* inhibition of lipid accumulation induced by oleic acid and *in vivo* pharmacokinetics of chitosan microspheres (CTMS) and chitosan-capsaicin microspheres (CCMS)

**DOI:** 10.1080/16546628.2017.1331658

**Published:** 2017-06-14

**Authors:** Sihui Wu, Haitao Pan, Sirong Tan, Chen Ding, Guidong Huang, Guihua Liu, Jiao Guo, Zhengquan Su

**Affiliations:** ^a^ Guangdong Food and Drug Vocational-Technical School, Guangzhou, China; ^b^ Key Research Center of Liver Regulation for Hyperlipidemia SATCM/Class III Laboratory of Metabolism SATCM, Guangdong TCM Key Laboratory for Metabolic Diseases, Guangdong Pharmaceutical University, Guangzhou, China; ^c^ School of Pharmaceutical Sciences, Sun Yat-sen University, Guangzhou, China; ^d^ Centre for Cellular & Structural Biology, Sun Yat-sen University, Guangzhou, China; ^e^ Shenzhen Center for Disease Control and Prevention, Shenzhen, China

**Keywords:** Chitosan microspheres, capsaicin-chitosan microspheres, distribution, lipid accumulation, obesity, FBS

## Abstract

Chitosan and capsaicin are compounds extracted from natural products and have been indicated to lower body weight and prevent fatty liver. However, their applications are limited by poor oral bioavailability, low compliance and some serious side effects. To solve these problems, we successfully prepared chitosan microspheres (CTMS) and chitosan-capsaicin microspheres (CCMS) in previous study. Therefore, in the present study, we evaluated the ability of CTMS and CCMS to eliminate lipid accumulation in hepatocytesand also characterized their pharmacokinetic parameters after administration. The results showed that the two microspheres could significantly reduce intracellular lipid accumulation and dose-dependently improve the triglyceride (TG) content in HepG2 cells. A pharmacokinetic study indicated that CTMS and CCMS were distributed in almost all of the measured tissues, especially liver and kidney, and that their absorption was better than those of chitosan and capsaicin. Simultaneously, the prolonged circulating half-lives, the lower clearance and higher plasma concentration of CTMS and CCMS showed that their bioavailability was effectively enhanced. All of the results indicated that the lipid accumulation inhibition of CTMS and CCMS was better than that of chitosan and capsaicin, and that these microspheres can be developed as preventive agents for fatty liver or obesity.

## Introduction

With the improvement in living standards, obesity has become an increasingly serious problem [1–3]. Although there have been several drugs developed for weight loss, including orlistat, fenfluramine and sibutramine, among others, most of them have been withdrawn from the market because of safety problems [4,5]. Therefore, Chinese medicine and phytochemical drugs have recently attracted much interest.

**Figure 1.**
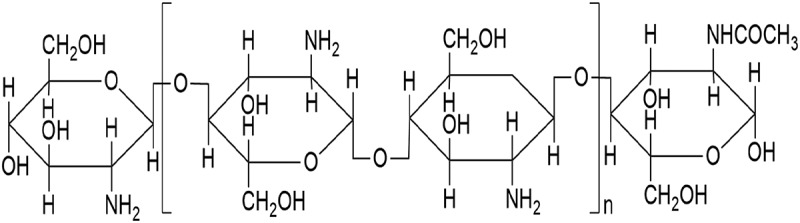
Chemical structure of chitosan (n ≥ 2).

Chitosan is polymer of glucosamine derived from the cell walls of some fungi and the exoskeleton of crustaceans, including shrimps, crabs, lobsters, and prawns (Figure 1). Because of its biocompatible and biodegradable properties, chitosan has been widely used in pharmaceutical research [6–9]. Currently, more human trials and animal experiments have demonstrated that chitosan can effectively lower blood lipid levels and body weight to normal levels [10–12]. However, its applications are limited by excessive nausea, vomiting, constipation, and the other side effects caused by large doses. We previously prepared chitosan microspheres (CTMS) and showed that their anti-obesity and lipid-lowering effects were more prominent than chitosan alone [13–16].

Capsaicin extracted from hot peppers can control the weight of people who are either overweight or suffering from obesity [17–19]. However, the pungency and poor oral bioavailability of capsaicin has made it difficult to be applied as an anti-obesity agent. To overcome these shortcomings, we developed capsaicin-chitosan microspheres (CCMS) by crosslinking capsaicin with chitosan via ionization and spray drying [20]. Additionally, CCMS can be made into enteric-coated tablets to further reduce the irritation of capsaicin. Previous anti-obesity experiments showed that CCMS was better than either capsaicin or CTMS for treating obesity. A possible explanation is that the smaller particle size helped capsaicin enter the bloodstream more easily and chitosan simultaneously promoted the absorption of capsaicin. Some related studies have indicated that the primary reason for improvements in the anti-obesity effects of CTMS and CCMS may due to improvements in their bioavailability [20].

To verify whether the pharmacokinetic parameters of chitosan were improved or not after preparing it as CTMS or CCMS, we labeled CTMS with fluorescein isothiocyanate (FITC) to track its tissue distribution and to further quantify it. Simultaneously, we quantified CCMS using an established high performance liquid chromatograph (HPLC) with capsaicin as the detection target.

Fatty liver diseases can be divided into two categories: alcoholic fatty liver disease (AFLD) and non-alcoholic fatty liver disease (NAFLD) [21]. NAFLD, which is a type of obesity-related metabolic disorder, is more commonly found in clinical practice and can seriously influence a patient’s quality of life [22,23]. Ongoing clinically therapeutic schedules for NAFLD include insulin sensitization agents, hypolipidemic drugs, anti-obesity pills, antihypertensive agents, cell protective agents, anti-inflammatory cytokine antioxidants, and other types of medications [21,24,25]. The present study firstly explored the therapeutic effects of CTMS and CCMS on NAFLD in a lipid-accumulation HepG2 cell model induced by oleic acid, which simulated the in-vitro pathological process of clinical NAFLD. The inhibition effects of CTMS and CCMS on intracellular lipid accumulation were also evaluated. This study demonstrated that CTMS and CCMS had potential value for the prevention and treatment of NAFLD.

## Materials and methods

### Animals

Male Sprague-Dawley (SD) rats (body weight 200 ± 20 g) were provided by the Medical Experimental Animal Centre of Guangdong Province (GMLAC, Guangzhou, China). All animal experimental protocols were approved by the Institutional Animal Care and Use Committee of Guangdong Pharmaceutical University (Guangzhou, China). The animals were housed in a specific pathogen free (SPF) standard room at 23–25°C with a relative humidity of 40–70% and a differential pressure no less than 10Pa under a constant day–night rhythm. The rats were given water ad libitum throughout the experiments. All animals were acclimated in the SPF room for at least one week prior to experimentation. The animals fasted for 12 hr with free access to water before treatments were given.

### Cell lines

The human hepatocellular carcinoma cell line HepG2 was kindly provided by the Institute of Chinese Medical Science of Guangdong Pharmaceutical University.

### Drugs and reagents

Chitosan (Lot, 10321A; viscosity, 200 mP/s; degree of deacetylation, 96.2%) was purchased from Shandong Aokang Biotech Ltd. (Shandong, China). CTMS and CCMS were prepared in our lab according to [16,20]. FITC was supplied by Sigma-Aldrich (St. Louis, MO, USA). HPLC grade methanol was provided by Honeywell International Inc. (Burdick and Jackson, Muskegon, MI, USA). Capsaicin was purchased from Wuhan Shengtianyu Ltd. (Wuhan, China) and its control from Tongtian Biotech Ltd (Shanghai, China). GIBCO Dulbecco’s modified Eagle’s medium (DMEM, Lot No. 1177237) was supplied by Invitrogen Co. Ltd. (USA). Thiazolyl blue tetrazolium bromide (3-(4,5-Dimethylthiazol-2-yl)-2,5-diphenyltetrazolium bromide – MTT, Lot No. M2128) was purchased from Sigma-Aldrich (St. Louis, MO, USA). Foetal bovine serum (FBS, Lot No. NWJ0473) was provided by Hyclone Co. Ltd. Other related reagents included Trypsin (Lot No. 0458) from Ameresco Co. Ltd.; Sodium oleate (Lot No. W76EC-OJ) from Tokyo Chemical Idustry Co. Ltd.; Oil red O staining solution (Lot No. 612121) from Zhuhai Beisuo Biological Technology Co. Ltd.; and a triglyceride measurement kit (Lot No. 125691) from Nanjing Jiancheng Bioengineering Institute, China. All other reagents were of analytical grade. Deionized, double glass-distilled water was used for chromatography.

### Fluorescence labelling of CTMS

#### Preparation for standard FITC curve

First, 1 mg/mL FITC in methanol solution was diluted with 0.1 M acetic acid solution (HAc) solution into a series of standard FITC solutions with 4.38, 8.76, 10.95, 13.14, 15.33, 19.71, 21.90, and 24.09 µg/mL. The absorbance (OD value) of each standard FITC solution at 443 nm was determined with a Mithras LB940 multimode microplate reader (Berthold Technology Co. Ltd., Germany).

#### The labelled CTMS (FITC-CTMS) and chitosan (fitc-chitosan)

CTMS and chitosan were dissolved in 20 mL of an 0.5% acetic acid solution with magnetic stirring, and the pH was adjusted to 7.5 with 1 M NaOH (the preparation method of CTMS was referenced to [16]). Then, 2 mL of a FITC dimethyl sulfoxide (DMSO) solution (1 mg/mL) was immediately added to the suspension while avoiding light. After 3 hrs of stirring in the dark, the target FITC-labeled precipitate was obtained. The precipitate went through a washing process with a methanol-water solution (centrifuged them once with 10,000 rpm for 15 min) until there was no absorbance in the supernatant at 443 nm. The resulting precipitate was collected and then dried at 40°C. The dry precipitate (FITC-CTMS and FITC-chitosan) was used in the animal experiments.

#### Optimization of labeling conditions (single factor experiment)

The precipitate above was dissolved with 0.1 M HAc solution with an FITC absorbance that was determined with a Mithras LB940 multimode microplate reader. The labeling efficiency was calculated according to the determined OD value with the formula:

### Labeling efficiency = FITC(μg)/FITC -labeled precipitate (μg)×100%

The labeling conditions influencing the labeling efficiency, which were the reaction time, concentration of CTMS, mass ratio of FITC and CTMS (or chitosan), pH and reaction temperature, were optimized. The ranges these influencing factors were chosen as the following:

*Reaction time*: 0.2, 0.4, 0.6, 1.0, 2.0, 3.0, 4.0, 5.0, and 6.0 hr, respectively;
*Concentration of CTMS*: 0.75, 2.25, 4.50, 6.25, and 9.00 mg/mL, respectively;
*Mass ratio of FITC and CTMS (or chitosan)*: 17, 22, 33, 67, and 200, respectively;
*pH*: 4.0, 5.4, 6.4, 7.5, 9.0, 10.5, and 12.0, respectively;
*Reaction temperature*: 10.0, 17.5, 25.0, 32.5, and 40.0°C, respectively.

#### Optimization of labeling conditions (the orthogonal experiment)

According to the single factor results, we proceeded with an orthogonal test using greater factors to prepare the FITC-CTMS and FITC-chitosan with high labelling efficiency. Simultaneously, three batches of FITC-labeled products were prepared in the replication tests to confirm the stability of the labeling conditions above.

### Distribution and expression of FITC-CTMS and FITC-chitosan

#### Animal experiment

Eighty male SPF SD rats (weight, 200 ± 20 g; age, 8 weeks) were used for animal experiments. All the protocols before administration were the same as the protocols described in the first section. The rats were divided into 16 groups (five rats per group) and 40 rats in eight groups received a dose of 450 mg/Kg FITC-CTMS by oral gavage, and the other 40 rats in eight groups received 450 mg/Kg FITC-chitosan in the same manner.

After administering the microspheres, the rats were sacrificed at 0, 1, 2, 4, 6, 12, and 24 hr for the FITC-CTMS and FITC-chitosan groups, respectively. The blood samples were immediately drawn from the orbital vein using a capillary after ether anaesthesia and centrifuged at 3,500 rpm for 10 min at 4°C. The serum was frozen immediately and stored at −20°C until further analysis. The heart, liver, kidneys, lungs, and spleen were also quickly excised, washed, and stored at −80°C until further analysis.

The other ten rats in the corresponding groups of FITC-CTMS and FITC-chitosan (five rats per group) were sacrificed at 0, 4, 8, 12, 24, and 36 hr after administration. Both urine and faeces samples were collected. The faecal samples were dried at 60°C and then powdered, and the urine samples were centrifuged. The urine supernatants and faeces powder samples were all stored at −80°C until extraction and analysis.

#### Preparation of biological samples

*Serum samples*: First, 200 μL hydrochloric acid (1 M) were added to 200 μL serum and mixed well. The homogeneous samples were centrifuged with 5,000 rpm at 4°C for 10 min, and the resulting supernatant was collected for further analysis.

*Tissues samples*: For tissues, 0.4 g of each tissue, including heart, liver, kidney, lung, and spleen, were homogenized completely in normal saline solution on ice. Then, 0.4 mL of the homogenate solution were added to 0.4 mL of hydrochloric acid (1 M) and mixed well. The suspension was centrifuged at 5,000 rpm at 4°C for 10 min, and the resulting supernatant was collected for further analysis.

*Urine and faecal samples*: The preparation protocols for urine samples and faecal samples were the same as the serum samples and tissues samples, respectively.

#### Obtaining a standard curve for FITC-CTMS and FITC -chitosan

To create a standard curve, 3.125 mg/mL FITC-CTMS in HAc solution was first diluted with 0.1 M HAc solution and 0.4 mL of the corresponding blank suspension samples (serum, tissue, urine, and faecal samples prepared as discussed in the section *‘*Distribution and expression of FITC-CTMS and FITC-chitosan – Preparation of biological samples) were added to the reaction to obtain a series of standard FITC-CTMS solutions including (1) standard 1 with 0.65, 1.30, 2.60, 5.21, 10.42, and 20.83 μg/mL (for measuring serum specimens); (2) standard 2 with 1.95, 3.91, 7.81, 15.63, 31.25, 46.88, 93.75, and 187.50 μg/mL (for measuring heart, liver, kidney, urine, and faeces samples); and (3) standard 3 with 0.49, 0.98, 1.95, 3.90, 7.81, 11.71, and 23.43μg/mL (for measuring of spleen and lung samples). The fluorescence intensity values of each standard were determined at 485 nm (excitation wavelength) and 535 nm (emission wavelength) with a Mithras LB940 multimode microplate reader.

Then, 2.813 mg/mL FITC-chitosan in HAc solution was diluted with 0.1 M HAc solution and 0.4 mL of the corresponding blank suspension samples (serum, tissues, urine, and faecal samples prepared as described in the section ‘Distribution and expression of FITC-CTMS and FITC-chitosan - Preparation of biological samples*’*) were simultaneously added to the reaction, which resulted in a series of standard FITC-chitosan solutions, including (1) standard 1 with 0.58, 1.17, 2.34, 4.68, 9.38, 18.75, 37.50, 56.25, and 112.50 μg/mL (for measuring serum specimens); (2) standard 2 with 1.75, 3.51, 7.03, 14.06, 28.13, 42.19, 84.37, and 168.75 μg/mL (for measuring heart, liver, kidney, urine, and faeces samples); and (3) standard 3 with 0.88, 1.75, 3.51, 7.03, 14.06, 21.09, 42.18, and 84.37 μg/mL (for measuring spleen and lung samples). The fluorescence intensity value of each standard was determined at 485 nm (excitation wavelength) and 535 nm (emission wavelength) with a Mithras LB940 multimode microplate reader.

#### Recovery, precision, and stability tests

*Recovery*: FITC-CTMS and FITC-chitosan were dissolved at high, middle, and low concentrations along with 0.1 M HAc solution (with three parallel samples for each concentration), and then each solution was processed according to the protocol in section *‘*Distribution and expression of FITC-CTMS and FITC-chitosan – Preparation of biological samples*’* to determine the recovery for each sample.

*Precision*: FITC-CTMS and FITC-chitosan were dissolved at high, middle, and low concentrations with 0.1 M HAc solution (with three parallel samples for each concentration). Each solution was processed and measured according to Section 'Distribution and expression of FITC-CTMS and FITC-chitosan – Preparation of biological samples' at each 3 hr period for within-day precision and for at three continuous days for day-to-day precision.

*Stability*: Three paralleled FITC-CTMS and FITC-chitosan serum and tissue homogenates were prepared as described in the section *‘*Distribution and expression of FITC-CTMS and FITC-chitosan - Preparation of biological samples', Distribution and expression of FITC-CTMS and FITC-chitosan: 2’ to investigate their stability over 6-hr period at 4°C.

### Distribution and expression of CCMS

#### Animal experiment

For the animal experiments, 132 male SPF SD rats (weight, 200 ± 20 g; age, 8 weeks) were used. The conditions in which the animals were raised were the same as described in the section ‘Distribution and expression of FITC-CTMS and FITC-chitosan - Animal experiment’ before the animals were divided into groups. The 132 rats were divided into 22 groups (six rats per group), and 66 rats in 11 groups were given a dose of 30 mg/Kg capsaicin by oral gavage, and the other 66 rats in the 11 groups were given 30 mg/Kg CCMS (30 mg/Kg of capsaicin was used to prepare CCMS as we described in our previous study) in the same manner.

After treatment, the rats were sacrificed at 0, 0.5, 1, 1.5, 2, 2.5, 3, 3.5, 4, 6, and 8 hr for all groups. The blood samples were immediately withdrawn from the orbital vein using a capillary after ether anaesthesia and centrifuged at 3,500 rpm for 10 min at 4°C. The serum was frozen immediately and stored at −20°C until further analysis. The heart, liver, kidneys, lungs, and spleen were also quickly excised, washed, weighed, and stored at −80°C until further analysis.

An additional ten rats in the corresponding groups for capsaicin and CCMS (five rats per group) were sacrificed at 0, 4, 8, 12, 24, 36, and 48 hr after treatment. Both urine and faecal samples were collected. The faecal samples were dried at 60°C and then powdered, and the urine samples were centrifuged. The urine supernatants and faeces power samples were all stored at −80°C until extraction and analysis.

#### Preparation of biological samples

##### Extracting solvents (via serum samples)

We chose different solvents as the candidates for the determination of samples in the future, including ethyl acetate, methanol, methanol-tetrahydrofuran (1:1), acetone-ethyl acetate (2:1), acetone-ethyl acetate (1:1), and acetone- ethyl acetate (1:2). The measured peak area of capsaicin was compared to the capsaicin standard, which confirmed the optimal solvents.

##### Samples

Either 0.2 g of tissue or 200 μL of serum samples were added to 200 μL of normal saline and then homogenized with a MICCRAD-1 homogenizer (ART Co. Ltd., Germany) or mixed well. The homogeneous serum and tissue samples were centrifuged with 5,000 rpm at 4°C for 10 min and the resulting supernatant was used for further analysis. Either 1.0 mL of serum or tissue homogenate and 5 mL of a suitable extracting solvent were added to 15-mL falcon tubes, mixed well for 2 min and centrifuged at 4,000 rpm for 15 min at 4°C. The organic layer was immediately transferred into another 15-mL falcon tube, and then dried with a HSC-24A nitrogen blowing concentrator (Zhengzhou Wobang Apparatus Co. Ltd., China). The residues were re-suspended with 150 μL of methanol (chromatographic grade) and were centrifuged at 12,000 rpm for 2 min at 4°C. The resulting supernatant was used for HPLC injection (10 μL per sample).

#### Quantification of capsaicin in biological samples

The samples were separated and quantified via HPLC in a Waters 2695–2998 system using a Diamonsil 250 mm*4.6 mm C18 column and a mobile phase consisting of methanol-sterile water (72/28, v/v) at a flow rate of 1.0 mL/min. All the samples to be tested were filtered through a 0.22 µm membrane, and the injection volume was 10 μL. The detection wavelength was 280 nm, and the peak position and area of capsaicin was identified based on the methodology assay and calibration curve generated from the standard compound of capsaicin. The preparation of the calibration curve involved the following steps: the blank suspension samples (serum, tissues and urine samples prepared as in the section *‘*Distribution and expression of CCMS - Preparation of biological samples’) were diluted with 0.1 M capsaicin solution to obtain a series of standard capsaicin solutions of 0.297, 0.891, 2.673, 5.346, 10.692, and 21.384 μg/mL, respectively. The relationships between the peak area (A) and concentration (C) were evaluated with linear regression analysis with a signal to noise ration ≥ 10. The samples for the methodology assay for capsaicin included: blank serum (or tissues, urine, and faeces), serum (or tissues, urine, and faeces) containing capsaicin (> 95.0%) and serum (or tissues, urine, and faeces) containing standard capsaicin (> 98.0%), which was prepared as described above.

#### Recovery, precision and stability tests

##### Recovery and precision

Capsaicin was dissolved in high, middle, and low concentrations with 1 ml of serum or tissue homogenate (three parallel samples were made for each concentration), and then handled and measured according to the section ‘Distribution and expression of CCMS - Quantification of capsaicin in biological samples’ for each recovery, within-day precision (3 hr) and day-to-day precision for three continuous days.

##### Stability

Three parallel capsaicin serum and tissue homogenate samples were prepared as described in the section ‘Distribution and expression of CCMS - Preparation of biological samples’ to investigate stability during two days at 4°C.

### In vitro inhibition of lipid accumulation induced by oleic acid

#### In vitro cytotoxicity assay

The cytotoxicity of CTMS and CCMS were evaluated in HepG2 cell lines using MTT (3-(4,5-dimethylthlthiazol-2-yl)-2,5-diphenyl tetrazolium bromide) assays. Briefly, 1.0 × 10^4^ per well of HepG2 cells were seeded in 96-well plates incubated in culture medium and DMEM with 10% FBS. After a 24 hr incubation in an incubator containing 5% CO2 at 37°C, the culture medium was removed and treated with a range of CTMS and CCMS concentrations, and the blank groups were simultaneously set up. The final concentrations were 1.0 × 10^6^, 1.0 × 10^5^, 1.0 × 10^4^, 1.0 × 10^3^, 1.0 × 10^2^, 1.0 × 10^1^, and 1.0 μg.L^−1^, respectively. Cell viability was detected at 48 hr postexposure when 20 μL of MTT (5.0 mg/mL) was added to the final concentration of 0.5 mg/mL, and then the cells were incubated for a further 4 hr at 37°C in the dark. Subsequently, the culture medium was removed, 150 μL of DMSO was added and the plate was shaken for 20 min on a cell oscillator to dissolve the formazan crystals. The OD value was read at a wavelength of 485 nm on a Mithras LB940 multimode microplate reader, and the cell survival rate (CSR) was calculated according to the following formula. Each experiment was repeated at least three times.

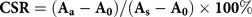

A_a_, A_s_, and A_0_ represent the absorbance of the experiment group, no-CTMS or no-CCMS control group, and the HepG2 cells blank group, respectively, in the formula.

#### Oil red o staining for HepG2 cells

For Oil red O staining, 2.5 × 10^5^ per well HepG2 cells were subcultured in six-well culture plates following a 24 hr incubation in the 37°C incubator containing 5% CO_2_ and the cells were subsequently divided into nine groups (with six parallel wells for each group): the blank (vehicle), model and berberine control group, and the CTMS and CCMS groups with high (1.0 × 10^6^μg.L^−1^), middle (1.0 × 10^5^μg.L^−1^), and low (1.0 × 10^4^μg.L^−1^) concentrations, respectively. The ingredients in each group are listed below

Vehicle: high glucose DMEM containing 10% FBS;
Model: high glucose DMEM containing 10% FBS and 0.2 mM (final concentration, same below) oleic acid;
Berberine control: high glucose DMEM containing 10% FBS, 0.2 mM oleic acid and 1 × 10^6^ μg.L^−1^ berberine;
CTMS: high glucose DMEM containing 10% FBS, 0.2 mM oleic acid and 1.0 × 10^6^ (high), 1.0 × 10^5^ (middle) and 3 × 10^4^μg.L^−1^ (low) CTMS;
CCMS: high glucose DMEM containing 10% FBS, 0.2 mM oleic acid and 1.0 × 10^6^ (high), 1.0 × 10^5^ (middle) and 1 × 10^4^μg.L^−1^ (low) CCMS.

After 24 hr of incubation, the culture medium was removed and washed with 1 × PBS (phosphate buffer) (pH7.4), and the experiment was conducted as follows: (1) the reaction systems were fixed for 20 min with 500 μl of 4% paraformaldehyde and then washed with 1×PBS (pH7.4) three times; (2) 500 μL of oil red O working solution were added to each well and stained for 15 min at room temperature; (3) the samples were fixed for 1 min with 60% isopropanol and immediately washed with 1 × PBS (pH7.4) three times until the backgrounds become transparent; (3) the cell morphology and its lipid droplet distribution were observed with an AX10 inverted fluorescence microscope (Carl Zeiss, Germany); (4) 500 μL of 60% isopropanol were injected into each well and then incubated for 15 min at room temperature; (5) after the extraction, 200 μL of each leaching liquor were injected into 96-well culture plates to determine the OD value with a Mithras LB940 multimode microplate reader at 485 nm.

#### Determination of the intracellular TG

For measuring TG, 5.0 × 10^4^ per well of HepG2 cells were inoculated in 24-well culture plates and incubated for 24 hr. All the experimental groups were the same as the groups described in the section ‘In vitro inhibition of lipid accumulation induced by oleic acid - Oil red O staining for HepG2 cells'. After 24 hr, the culture solution was removed and washed with precooled 1 × PBS (pH7.4) three times. The experiment was conducted as follows: (1) added 1 × 10^7^ cells/mL precooled RIPA lysis buffer into each system for approximately 10 min to produce sufficient lysis; (2) scraped the pyrolysis products gently with cell scrapers and transferred them into 1.5-mL Eppendorf tubes; (3) the samples were shocked for 30 min and then centrifuged at 12,000 rpm on ice for 15 min before the supernatants were collected and 10 μL of each one were used for protein quantification. Total proteins in HepG2 cells were quantified with the Pierce BCA protein assay kit (Thermo Fisher Scientific Inc., USA), and the remaining supernatants were used for determining intracellular TG according to triglyceride determination kits (Nanjing Jiancheng Bioengineering Institute, China).

### Data analysis

The pharmacokinetic parameters were calculated by 3p97, and the area under the blood concentration-time curve (AUC) and half-life (*t*_1/2_) of the administered capsaicin were calculated using standard formulae. The results are expressed as the mean±SEM, and comparisons between the groups were made with an unpaired Student’s t-test. Differences were considered to be statistically significant if *p *< 0.05.

## Results

### Fluorescence labeling of CTMS

To quantify the labeled CTMS (FITC-CTMS), we prepared the standard curve of FITC first. The regression equation was y = 0.0339 x +0.0941, and its correlation coefficient (R^2^) was 0.9993, which were suitable for the quantitative analysis of FITC-CTMS.

**Figure 2.**
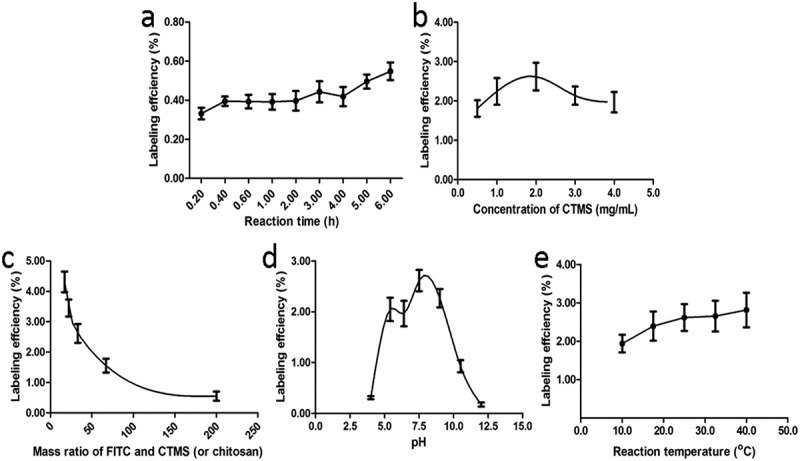
The single labeling factors influencing the labeling efficiency included the reaction time (a), concentration of CTMS (b), mass ratio of FITC and CTMS (c), pH (d), and reaction temperature (e).

To obtain stable FITC-CTMS for treatment, several influencing factors, including the reaction time, concentration of CTMS, mass ratio of FITC and CTMS (or chitosan), pH and reaction temperature were tested (Figure 2). The reaction time had little effect on the labelling efficiency (Figure 2(a)), although the labeling efficiency increased slowly after 4 hr, we also chose 3 hr as the reaction time in the following orthogonal analyses because the microsphere structure of CTMS may have changed over time. The other four single factors, including the concentration of CTMS, mass ratio of FITC and CTMS (or chitosan), pH and reaction temperature, had a greater influence on the labelling results (Figure 2(b-e)). When the concentration of CTMS was 2 mg/mL (Figure 2(b)), and pH 7.5 (Figure 2(d)), we obtained the highest labeling efficiency. According to these results, we set CTMS concentrations 1, 1.5, 2 mg/mL and at pH 5.5, 7.5, 9.5 as the three orthogonal levels, respectively. Figure 2(c) shows that the labeling efficiency decreased as the mass ratio of FITC and CTMS (or chitosan) increased. The high ratio of FITC and the labeled target can greatly alter the surface morphology of CTMS. Meanwhile, the lower ratio would have affected in vivo detection, so we chose CTMS:FITC 100:1.5, 100:3, and 100:4.5 as the conditions for the orthogonal optimization. For reaction temperature, the lower value will be bad for the reaction and the higher value will affect the structural stability of CTMS (Figure 2(e)). Therefore, we chose the lower temperature while meeting the desired labelling efficiency. In the study, we set 15, 25, and 35°C as the orthogonal levels.

The single factor experiment above indicated that the CTMS concentration, mass ratio of CTMS and FITC (CTMS:FITC), pH, and reaction temperature are the key factors for labeling (Table 1). Therefore, we chose the key factors above for the orthogonal analysis (Table 1) and led the best conditions for labeling include: CTMS:FITC = 100:1.5; pH7.5; CTMS concentration 1.5 mg/ml and a reaction temperature of 25°C. According to the optimized conditions, we obtained FITC-CTMS with a labeling efficiency of 2.2% (e.g. 2.2 mg of FITC per 100 mg of FITC-CTMS).

**Table 1.** Orthogonal analysis of CTMS labelling (*p < 0.05, **p < 0.01) including the factors and levels (A), the arrangement and results of orthogonal experiments (B), and the variance analysis (C).

Levels	Factors			
	(A) pH	(B) Temperature (°C)	(C) Ratio of CTMS:FITC	(D) Concentration of CTMS (mg/ml)
A				
1	5.5	15	100:1.5	1
2	7.5	25	100:3	1.5
3	9.5	35	100:4.5	2

No.	A	B	C	D	Label efficiency (%)
B					
1	1	1	1	1	0.05
2	1	2	2	2	0.43
3	1	3	3	3	1.92
4	2	1	2	3	1.49
5	2	2	3	1	2.65
6	2	3	1	2	0.60
7	3	1	3	2	0.97
8	3	2	1	3	0.75
9	3	3	2	1	0.34
K_1_	0.80	0.84	0.47	1.01	
K_2_	1.58	1.28	0.75	0.68	
K_3_	0.69	0.95	1.85	1.39	
R	0.89	0.44	1.38	0.72	

Factors	SS	f	F	F*	Significance
C					
A	4.04	2	2.02	518.44	**
B	1.09	2	0.54	139.68	*
C	9.89	2	4.94	1268.5	**
D	2.29	2	1.15	294.03	*

### Distribution and expression of FITC-CTMS and FITC-chitosan

#### Preparation for standard curve of FITC-CTMS and FITC-chitosan

The regression equations, correlation coefficients, and linear ranges of FITC-CTMS and FITC-chitosan in serum, heart, liver, kidney, spleen, lung, urine, and faeces are shown in Table 2, and all R^2^ for which the values are greater than 0.9900, met the requirements for determination of biological samples. Simultaneously, the results of recovery, precision, and stability tests demonstrated that all of the recoveries were greater than 75%, the intra-day and inter-day precision were 1.78%–8.10% and 4.36%–8.63%, and the RSD values of 6 hr stability for all of the samples were less than 10%, which met the demands of the analysis.

**Table 2.** The regression equations, R^2^ and linear ranges of FITC-CTMS (A) and FITC-chitosan (A) in different samples.

Sample	Regression equation	R^2^	Linear range (μg/mL)
A			
Serum	y = 96.29x + 413.72	0.9935	1.30~20.83
Heart	y = 58.90x + 1238.41	0.9964	1.95~187.50
Liver	y = 95.32x + 1880.54	0.9981	1.95~187.50
Spleen	y = 126.44x + 450.51	0.9955	0.98~23.43
Lung	y = 108.96x + 463.63	0.9960	0.98~23.43
Kidney	y = 72.84x + 2712.18	0.9970	1.95~187.50
Urine	y = 90.29x + 313.72	0.9932	1.95~187.50
Faeces	y = 86.23x + 410.77	0.9975	1.95~187.50
B			
Serum	y = 241.64x + 679.53	0.9952	1.17~112.50
Heart	y = 218.01x + 827.79	0.9962	1.75~168.75
Liver	y = 193.23x + 2937.70	0.9940	1.75~168.75
Spleen	y = 185.31x + 714.29	0.9972	0.88~84.38
Lung	y = 193.44x + 568.44	0.9931	0.88~84.38
Kidney	y = 211.59x + 3329.65	0.9980	1.75~168.75
Urine	y = 225.67x + 578.34	0.9965	1.75~168.75
Faeces	y = 203.63x + 781.12	0.9901	1.75~168.75

#### Distribution and expression

**Figure 3.**
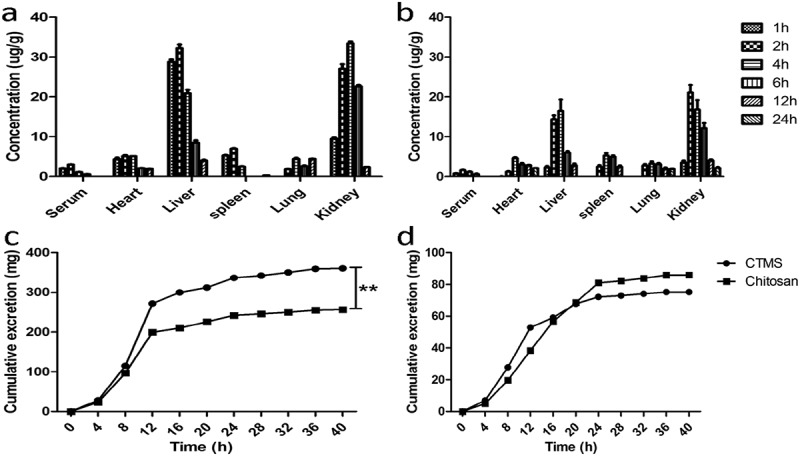
Distribution and expression of CTMS and chitaosan in SD rats after treatment. The 24 hr distribution of CTMS (a) and chitosan (b), as well as the urinary (c) and faecal (d) expression of CTMS and chitosan.

After oral administration of either FITC-CTMS or FITC-chitosan, the 24 hr distribution of CTMS and chitosan in the serum, liver, heart, spleen, lungs and kidneys are shown in Figure 3(a-b), which indicates that the absorption of FITC-CTMS was better than FITC-chitosan, and the highest concentration of FITC-CTMS occurred at 2 hr after administration. After absorption, FITC-CTMS and FITC-chitosan were distributed in all of the tissues above, and especially in liver and kidneys. However, after 24 hr, we could not determine the FITC-CTMS and FITC-chitosan in any of the above tissues, which suggested that the FITC-CTMS and FITC-chitosan could be quickly cleared from the body without long-term accumulation.

The urinary and faecal expression of FITC-CTMS and FITC-chitosan are shown in Figure 3(c–d), which indicated that the two FITC-labeled products are mainly expressed within 8–24 hr (255 μg and 359 μg via urine, and 86 mg and 75 mg via faeces, respectively). Therefore, only a small quantity of FITC-CTMS and FITC-chitosan was absorbed and a large proportion was excreted via faeces along with better absorption of FITC-CTMS.

### Distribution and expression of CCMS

#### Methodology and standard curve of CCMS

The methodology of capsaicin in serum, heart, liver, spleen, lung, kidney, urine, and faeces was investigated as described in the section ‘Distribution and expression of CCMS: 3’). The results indicated that the retention time of capsaicin in serum and tissues was 10.5 min, and also there were no peaks of endogenous components around the targeted capsaicin peak, which met the determined requirements for capsaicin in CCMS.

The regression equations, correlation coefficients and linear ranges of CCMS in the serum, heart, liver, kidneys, spleen, lung, urine, and faeces are shown in Table 3 and the R^2^ values greater than 0.9900, met the requirements of determination for biological samples. Simultaneously, the precision and stability tests demonstrated that all of the RSD values of intra-day, inter-day precision and two-day stability were less than 10%, which also met the requirements of the analysis.

**Table 3.** The regression equations, R^2^ and linear ranges of CCMS in different samples.

Sample	Regression equation	R^2^	Linear range (μg/mL)
Serum	y = 47508x + 825.43	0.9995	0.297~21.384
Heart	y = 46721x + 1238.42	0.9994	0.297~21.384
Liver	y = 49232x + 1880.56	0.9991	0.297~21.384
Spleen	y = 48621x + 450.51	0.9955	0.297~21.384
Lung	y = 42381x + 1463.63	0.9990	0.297~21.384
Kidney	y = 47587x + 712.18	0.9992	0.297~21.384
Urine	y = 41366x + 1228.10	0.9994	0.297~21.384
Faeces	y = 41225x + 1139.97	0.9993	0.297~21.384

Additionally, in the recovery assay, we first optimized the best extraction solvent for the next determination according to the recoveries of the different solvents described in the section ‘Distribution and expression of CCMS - Preparation of biological samples’, which are listed in Table 4. Better and more stable recovery occurred when using acetone-ethyl acetate (1:1). Therefore, we chose acetone-ethyl acetate (1:1) as the solvent for additional experiments. Simultaneously, all of the recoveries of CCMS in serum, heart, liver, spleen, lung, kidney, urine and faeces were greater than 75%, which met the requirements for analysing biological samples.

**Table 4.** Optimization of the extraction solvent (shown as recovery).

Solvents	Low	Middle	High
Ethyl acetate	66%	70%	65%
Methanol-tetrahydrofuran (1:1)	70%	67%	65%
Methanol –trichloromethane (1:1)	70%	72%	71%
Methanol	A great deal of impure peaks		
Acetone- ethyl acetate (2:1)	80%	82%	78%
Acetone -ethyl acetate (1:1)	81%	80%	83%
Acetone -ethyl acetate (1:2)	82%	81%	83%

#### Drug concentration–time curve and pharmacokinetic parameters

**Figure 4.**
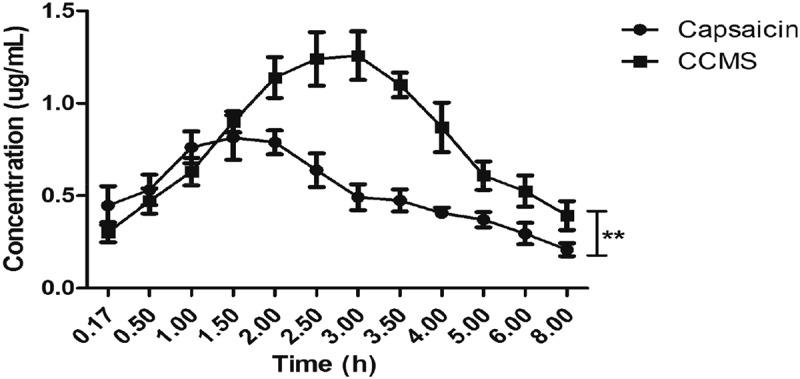
The mean drug concentration–time curve of capsaicin and CCMS.

The plasma concentration of capsaicin and CCMS were calculated with WinNonlin V4.0.1, which indicated that the pharmacokinetic processes of these molecules had the two-compartment model. The mean drug concentration–time curves and pharmacokinetic parameters of capsaicin and CCMS are shown in Figure 4 and Table 5, respectively. The maximum concentration (Cmax) and AUC of CCMS were higher compared to capsaicin, which suggested that the absorption and relative bioavailability of capsaicin improved after capsaicin was incorporated into CCMS.

**Table 5.** Pharmacokinetic parameters of capsaicin and CCMS after oral administration (n = 6).

Parameters	Units	Capsaicin	CCMS
**T_max_**	hr	1.33 ± 0.26	2.75 ± 0.27
**C_max_**	ng·mL^−1^	814.60 ± 59.47	1257.67 ± 99.85
**α_HL**	hr	0.30 ± 0.10	0.97 ± 0.17
**β_HL**	hr	2.22 ± 0.47	3.48 ± 0.53
**AUC_0→t_**	hr·ng·mL^−1^	3830.52 ± 667.65	5848.41 ± 754.81
**AUC_0→∞_**	hr·ng·mL^−1^	5015.38 ± 706.02	7262.58 ± 863.50
**CL_F**	mL·hr^−1^	5003.57 ± 616.28	3478.29 ± 452.22

*Notes*: CL = clearance; T_max_ = time to maximum concentration

#### Distribution and expression of capsaicin and CCMS

**Figure 5.**
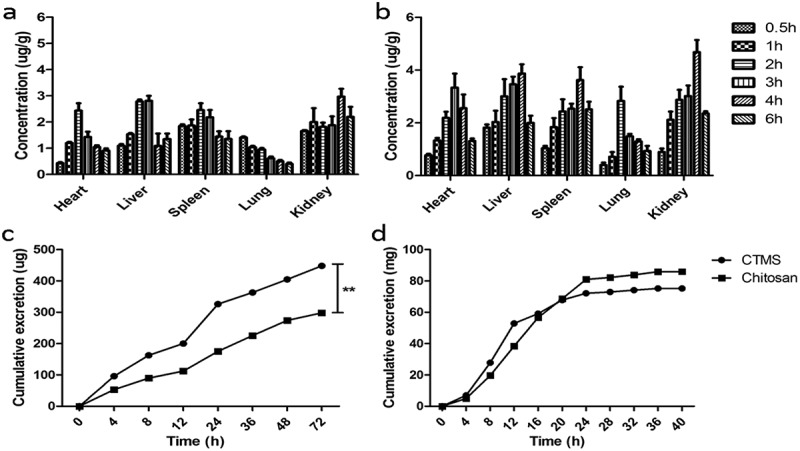
Distribution and expression of capsaicin and CCMS in SD rats after treatment. The 6 hr tissue distribution of capsaicin (a) and CCMS (b), as well as the 72 hr urinary (c) and faecal (d) excretion of capsaicin and CCMS.

The 6 hr in vivo distributions of capsaicin and CCMS are shown in Figure 5(a-b), which indicates that they were widely distributed in various organs. Considering the weight of these organs, most of the capsaicin was distributed in the liver and kidneys. Notably, after CTMS was loaded into CCMS, the concentrations of CCMS in different tissues were promoted through an increase in its absorption. The 72 hr expression of capsaicin and CCMS in urine and faeces are illustrated in Figure 5(c-d). For urine, they appeared for 72 hr and their fastest expression rates occurred within 4 hr. For faeces, the peak occurred at 12–24 hr and the capsaicin and CCMS could not be detected after 48 hr. Additionally, a significant amount of capsaicin was excreted through the urine while only a little was excreted in the faeces. Most importantly, the content of CCMS in urine or faeces after oral administration was lower compared to capsaicin.

### In vitro inhibiting effects of CTMS and CCMS on oleic acid-induced lipid accumulation in HepG2 cells

**Figure 6.**
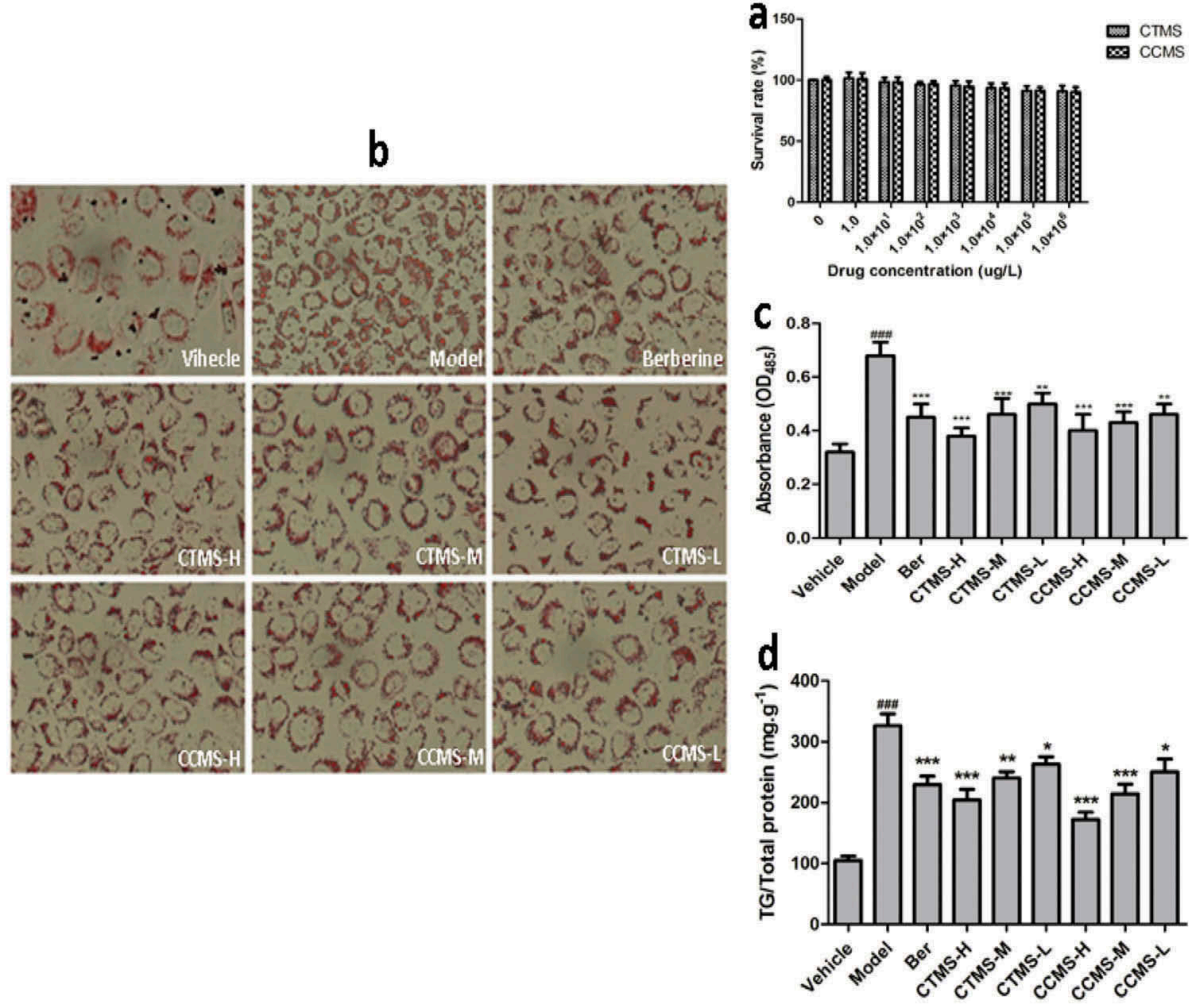
In vitro inhibiting effects of CTMS and CCMS on oleic acid-induced lipid accumulation in HepG2 cells. (a) The MTT assay results (x ± s, n = 6); (b) Oil red O staining maps of HepG2 cells; (c) Lipid accumulation inhibiting results; (d): Inhibiting effects on the TG content. Note: Vehicle: stained with only high glucose DMEMcontaining 10% FBS; Model: stained with high glucoseDMEM containing 10% FBS and 0.2mM(final concentration,same below) oleic acid; Ber (Berberine, positive):1.0 × 106 μg L−1 berberine; CTMS-H: 1.0 × 106 μg L−1715 CTMS; CTMS-M: 1.0 × 105 μg L−1 CTMS; CTMS-L:1.0 × 104 μg L−1 CTMS; CCMS-H: 1.0 × 106μg L−1CCMS; CCMS-M: 1.0 × 105μg L−1 CCMS; CCMS-L:1.0 × 104 μg L−1 CCMS. (###p < 0.001 when compared tothe control; *p < 0.05,**p < 0.01,***p < 0.001 when com-720 pared with the model).

We first investigated the HepG2 cell cytotoxicity of CTMS and CCMS in different concentrations using the MTT assay to choose the best action concentrations. The results are shown in Figure 6(a). Although the cell viability gradually decreased, the concentrations of CCMS and CTMS increased, and the cell viability were more than 90% when the concentrations were all less than 1.0 × 10^6^μg.L^−1^. Therefore, we chose 1.0 × 10^6^ μg.L^−1^, 1.0 × 10^5^ μg.L^−1^ and 1.0 × 10^4^ μg.L^−1^ as the high, middle, and low doses for the further activity assays of CCMS and CTMS, respectively.

The oil red O staining results are displayed in Figure 6(b). HepG2 cells in the induced group contain a high amount of red lipid droplets fused to each other, which indicated that the HepG2 cell lipid accumulated model was successfully established. HepG2 red lipid droplets show the dose-dependent trend that was characterized by decreasing number and volume as well as shallow colour. Compared to the induced group, the CTMS and CCMS middle and low concentration groups showed obviously improved lipid accumulation and alleviated lipid fusion in the HepG2 cells, and the CTMS and CCMS high concentration groups had more significant effects to the ones described above. The absorbance of HepG2 cells in each group are shown in Figure 6(c) and were measured after the process of oil red O staining and extraction with isopropanol. It is observed that there is a significantly increasing lipid accumulation in HepG2 cells in the model group compared to blank (*p *< 0.001), and the effects of CTMS and CCMS in the high and middle concentration groups on the reduction of lipid accumulation are comparable to those of the chitosan-positive control group.

The data of TG contents in different groups are shown in Figure 6(d). There was significantly increasing TG content in the model group compared to the blank control (*p *< 0.001), which indicated that the HepG2 lipid accumulation model was successfully established. The high and middle doses of CCMS and the high dose of CTMS effectively reduced the TG content (*p* < 0.001) compared to the model. An extremely significant difference existed between CTMS at the middle concentration and the model group (*p *< 0.01), and there were also significant differences between CTMS, CCMS, and the model groups. Notably, the decrease in TG accumulation was linearly correlated with the increase of CTMS and CCMS concentration in the range from 1.0 × 10^4^ to 1.0 × 10^6^ μg.L^−1^. Therefore, for CTMS and CCMS, 1.0 × 10^6^ μg.L^−1^ were determined to be the most potent suppressive concentration of TG content in HepG2 cells.

## Discussion

Due to safety concerns, it has been difficult to develop anti-obesity drugs. Therefore, natural medicine is a good choice for treating obesity. Currently, chitosan has been used as an important auxiliary material for pharmaceutic preparation and drug delivery systems [26,27]. However, when pharmacists only used served chitosan as the pharmaceutic adjuvant, they found that chitosan manifested ideal pharmaceutical bioactivities, such as neuroprotection, anti-cancer, antibacterial, anti-inflammatory, hypoglycaemia, antioxidants, and liver protection, among others [28]. However, its applications are limited due to excessive nausea, vomiting, constipation, and other side effects caused by large doses. We previously prepared chitosan microspheres (CTMS) and showed that their anti-obese and lipid-lowering effects were more prominent compared to chitosan alone [13–16].

To investigate the bio-distribution of chitosan in animals, radioisotope labeling and fluorescence labeling were selected to detect the presence of chitosan in the bodies. Some of the labeling methods used involve amine reactive compounds, such as FITC and 9-anthraldehyde, which can react with the amines of chitosan. Therefore, FITC is the most widely used fluorescent marker [29]. In the study, we used FITC-CTMS with a labeling efficiency of 2.2% after optimizing the reaction conditions.

The bio-distribution of FITC-chitosan and FITC-CTMS showed that the two main organs that contained these compounds were the kidneys and the liver, which appeared to be the primary target and that chitosan and CTMS may lower lipid levels by affecting hepatic functions. Our previous study indicated that the anti-obesity and fatty liver preventive performances of CTMS and CCMS were better than chitosan and capsaicin alone [30]. The reason is that the absorption of chitosan was improved when it was prepared as CTMS, which was shown in the present study.

Other researchers observed that the absorption of chitosan depended on its mean molecular weight (Mw). The lower the Mw was, the more absorption that occurred. Zeng et al. studied the absorption and distribution of four chitosan with different molecular weights in mice after oral administration [31], which suggested that the water solubility and Mw significantly influenced the absorption and distribution of chitosan. Chitosan with high water solubility and low Mw maintained a high drug concentration in all of the tested tissues and simultaneously had a long half-life. The same conclusion was reached by Chae et al. [32], and, most importantly, the smaller Mw and particle size of CTMS made it more effective, which was also verified in this study.

Capsaicin has shown some effects for controlling body weight by decreasing energy intake, adipose tissue weight, and serum triglyceride levels through stimulation of lipid mobilization [18,19,33,34]. Joo et al. demonstrated that thermogenesis and the proteins involved in lipid metabolism were markedly altered after capsaicin treatment in white adipose tissue [35]. Belza et al. observed that capsaicin supplements can increase the four-hour thermogenesis by 90kJ compared to placebo [36]. This effect was maintained for eight weeks and accompanied by a slight reduction in fat mass. However, the use of the capsaicin for oral administration was restricted due to its pungency. The microsphere delivery system was implemented for drug delivery because it can not only enhance the absorption of the drug but reduce adverse stimuli [37,38]. The CCMS we prepared had better weight-control results than capsaicin alone and also improved administration compared to capsaicin alone, which indicated that the absorption of capsaicin was enhanced and there was a simultaneous sustained release effect after capsaicin was prepared as CCMS.

Considering the results of the pharmacokinetics, biodistribution, and excretion rates, we observed that although the majority of capsaicin was absorbed within the body, only a small amount of it was detected, which showed that capsaicin can readily undergo the first-pass effect during absorption. Suresh et al. confirmed that although 94% of capsaicin was absorbed, the plasma concentration was also low [38]. Kawada observed that capsaicin was readily absorbed after oral administration, but the capsaicin was almost completely metabolized in the liver before reaching general circulation [39]. The metabolism of capsaicin has been reported to be similar in human, rat, and dog microsomes [40]. Researchers believed that cytochrome P_450_ enzymes were primarily responsible for the metabolism of capsaicin [40]. In this study, we prepared a type of CCMS encapsulating capsaicin in the chitosan microsphere and observed that the first-pass effect was reduced based on the measured AUC of capsaicin (Table 5). A possible explanation was that the gel formation of chitosan reduced the metabolism in the intestines, which indicated CCMS could be developed into a new type of anti-obesity drug.

NAFLD has gradually become a widespread and serious disease since the first official report of NAFLD was published in 1958. Now, the average incidence of NAFLD is approximately 20% with a yearly increase. As the leading cause of hepatic dysfunction worldwide, the typical characteristics of NAFLD includes intracellular lipid accumulation in hepatocytes [41]. The main liver lipid accumulating of NAFLD patients was TG, which resulted from an imbalance between lipid synthesis and transformation [22,23]. Genetic factors combined with the external environment and metabolic stress ultimately results in the pathogenesis of NAFLD, and the current theory for the molecular mechanisms of NAFLD is the ‘two-hit theory’ proposed by Donati and Diehl et al. [42,43]. HepG2 cells have been successfully used to establish a fatty liver cell model due to their stable cell characteristics, such as easy cultivation, which can be used to screen for preventive and therapeutic drugs and simultaneously explore fatty liver pathogenesis [44].

Long-chain fatty acids can form intracellular lipid droplets leading to lipid accumulation. As the foremost component of long-chain free fatty acids, oleic acid can be selected to establish lipid accumulation model in hepatocytes, which is also relevant to the pathophysiological process of NAFLD. Establishing a hepatocyte lipid accumulation model can be achieved through induction by 0.1 to 2 mmol.L^−1^ oleic acid for 24 hr in HepG2 cells [30,45]. The level of intracellular lipid accumulation is positively correlated with the oleic acid concentration, which can be measured using the standard curve method [46].

In the present study, we selected oleic acid as the 24 hr continuous inducer to establish a lipid accumulating model and further evaluated the roles of CTMS and CCMS for eliminating hepatic lipid accumulation. The conventional indexes of NAFLD, such as the TG content and lipid droplets in hepatocytes, were detected. The results showed that CTMS and CCMS can effectively inhibit the TG content and lipid accumulation dose-dependently, which were also better than chitosan alone.

Overall, all of the results indicated that the two microspheres, CTMS and CCMS, can significantly reduce intracellular lipid accumulation and dose-dependently improve the TG content in hepatocytes better than chitosan and capsaicin alone. The reason for this improvement is the enhanced absorption and bioavailability that occurs when chitosan was prepared as CTMS and CCMS. Therefore, CTMS could be developed as preventive agents for fatty liver disease or obesity.
